# Supplementary figures and images for: A novel cyclic peptide (Naturido) modulates glia–neuron interactions in vitro and reverses ageing-related deficits in senescence-accelerated mice
Source: PLoS One. 2021 Jan 27;16(1):e0245235. doi: 10.1371/journal.pone.0245235 (PMC7840003; doi:10.1371/journal.pone.0245235)

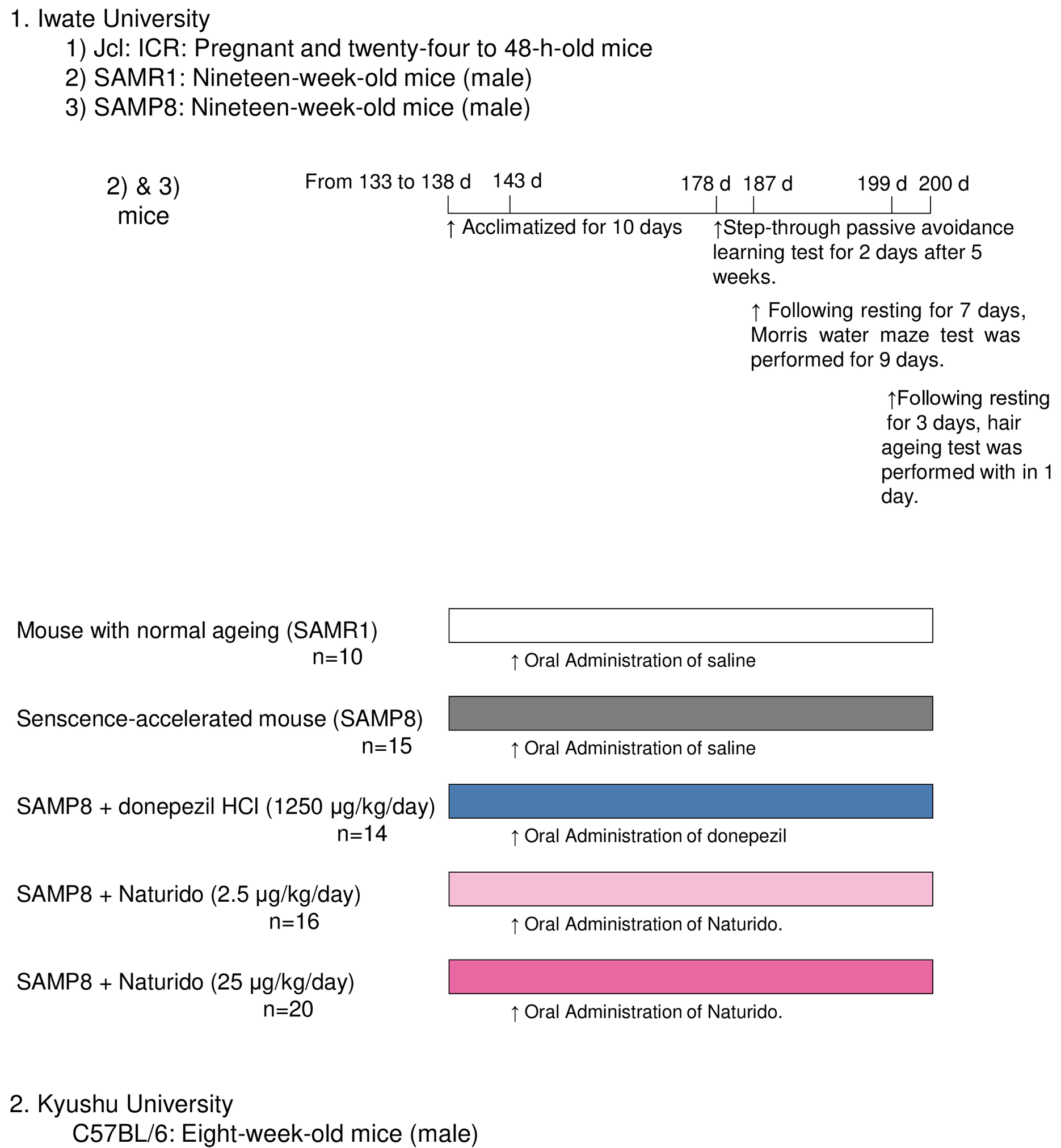

Supplement: S1 Fig — (TIF) [file pone.0245235.s001.tif]

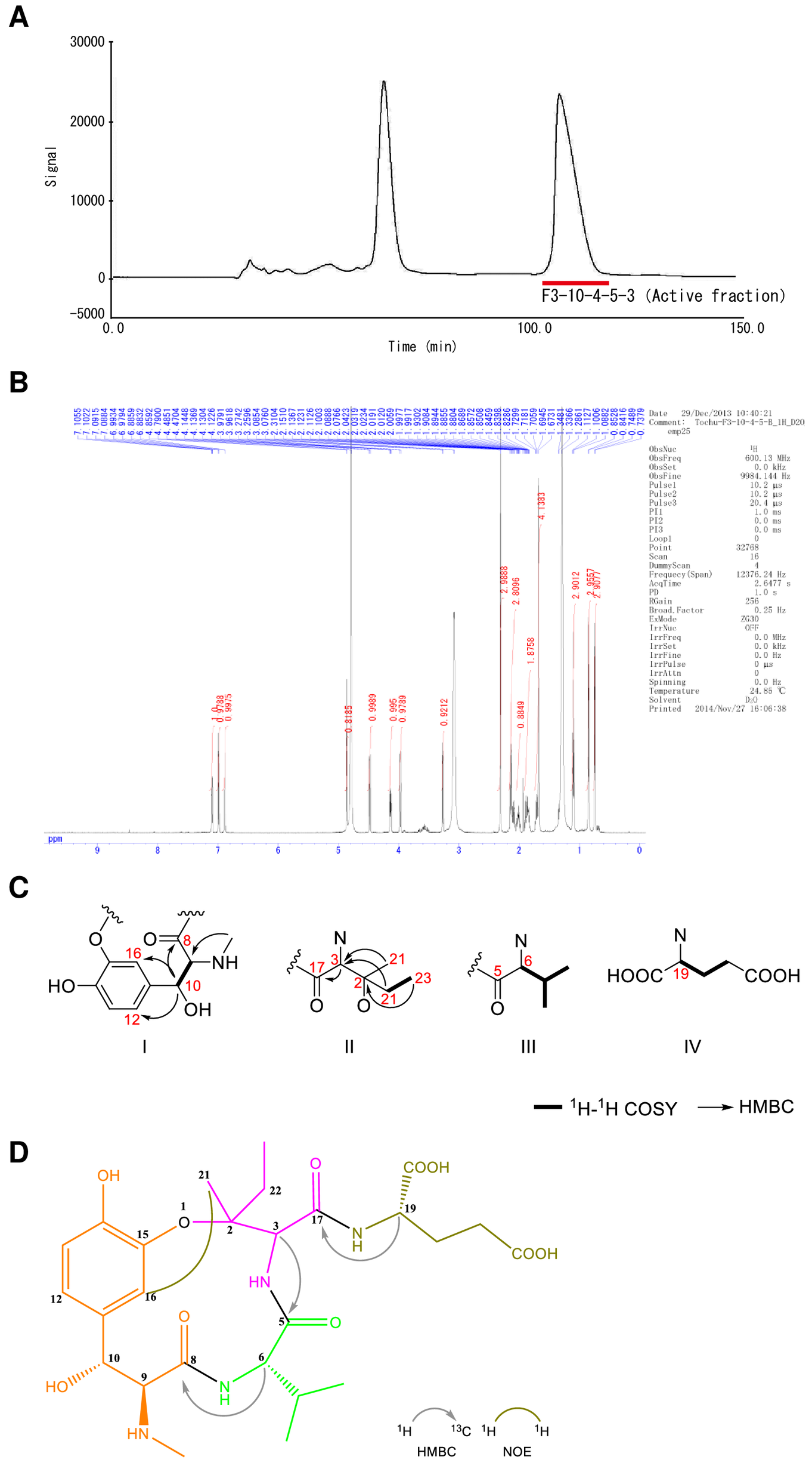

Supplement: S2 Fig — (A) After purification, the structure of F3-10-4-5-3 demonstrating astrocyte proliferation was considered a bis (diethylamine) salt ([α] D −21.6 (c 0.18, H2O)). The molecular formula for @ was established as C26H38N4O10. The parameters were as follows: highresolution electrospray ionization mass spectroscopy [HRESIMS]: fast atom bombardment (FAB) negative; matrix: glycerol; high-resolution mass spectrometry (HRMS) (FAB) m/z (M-H)-: calculated for [C26H37N4O10-H]-, 565.2510, found, 565.2512. The 1H NMR spectrum (in D2O, (B) showed resonances for six methine protons (δH 4.80, 4.75, 4.12, 4.03, 3.88, and 1.84 ppm), three methyl protons (δH 2.72, 1.67, and 1.03 ppm), characteristic upfield methyl groups (δH 0.71 and 0.81 ppm), and three aromatic protons of a 1,2,4-trisubstituted benzene (δH 7.15, 7.04, and 6.97 ppm). The 13C NMR spectrum showed five carbonyl carbons (δC 184.9, 181.0, 176.2, 173.9, and 173.0 ppm), six aromatic carbons (δC 153.5, 145.1, 134.6, 125.2, 124.2, and 121.4 ppm), and 15 carbons (δC 88.2, 77.6, 74.5, 62.4, 62.1, 58.4, 37.1, 36.4, 35.1, 31.8, 31.0, 23.3, 21.1, 20.7, and 10.4 ppm) in the aliphatic region. Further analysis of 1D and 2D NMR data indicated the 2 presence of four substructures (C). The structure of I was established to be β-hydroxy-3,4-dihydroxyphenylalanine by heteronuclear multiple bond correlation (HMBC) analysis, which indicated a correlation of H-10 with C-12, C-16 and C-8 and of 9-NMe with C-9. Partial structure II was revealed as a hydroxyl isoleucine moiety on the basis of the HMBC correlations of both H-21 and H-22 with C-3 and of H-23 with C-2 as well as the 13C chemical shift at C-2 (δC 85.6 ppm). Partial structures III and IV were rapidly classified as valine and glutamic acid, respectively, by 1H-1H correlation spectroscopy (COSY). (D) Cyclic peptide consisting of 4 amino acids (N-methyl-β-hydroxy DOPA, valine, β-hydroxyleucine, and glutamic acid). (TIF) [file pone.0245235.s002.tif]

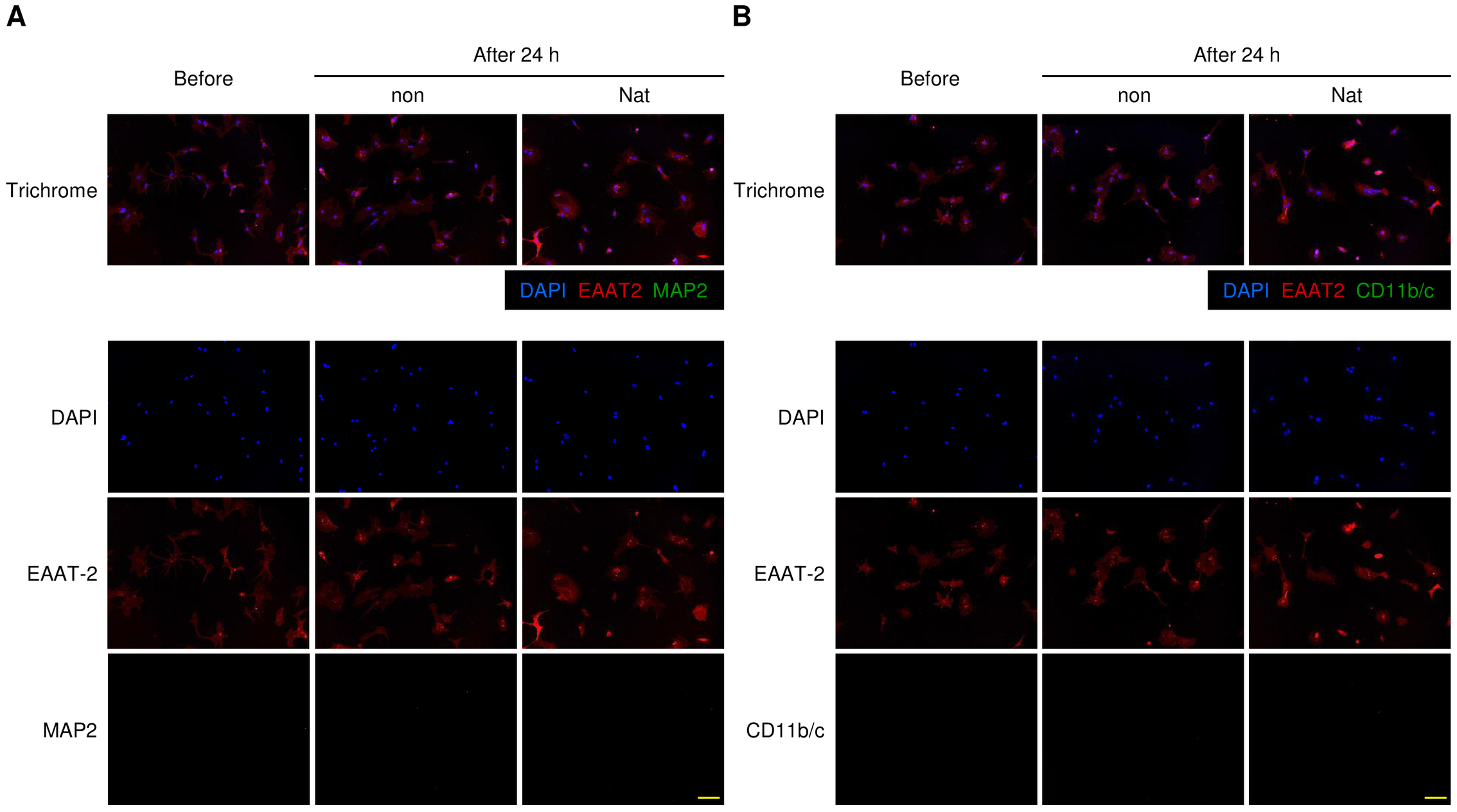

Supplement: S3 Fig — According to the sections of cell culture experiments and identification of primary cultured astrocytes in “materials and methods”, the astrocytes with a density of 5.6 × 10^3^ cells/cm2 were prepared and the cells were exposed to Naturido (25 μM) for 0 or 24 h under of 37°C and 5.0% CO2. LG-D-MEM (0% FBS) without the addition of Naturido was used as a control. Histochemical observations were carried out using DAPI for nuclear staining, anti-MAP2 for a neuronal marker, anti-CD11b/c for a microglia marker, and anti-EAAT-2 for an astrocyte marker. (A) Histochemical observations (left panel) of anti-MAP2 in the primary cultured astrocytes (before, 0 h, 24 h treated with Naturido) compared to the stains of trichrome, DAPI and anti-EAAT-2. (B) Histochemical observations (right panel) of anti-CD11b/c in the primary cultured astrocytes (before, 0 h, 24 h treated with Nautrido) compared to the stains of trichrome, DAPI and anti-EAAT-2. Scale bar = 100 μm. (TIF) [file pone.0245235.s003.tif]

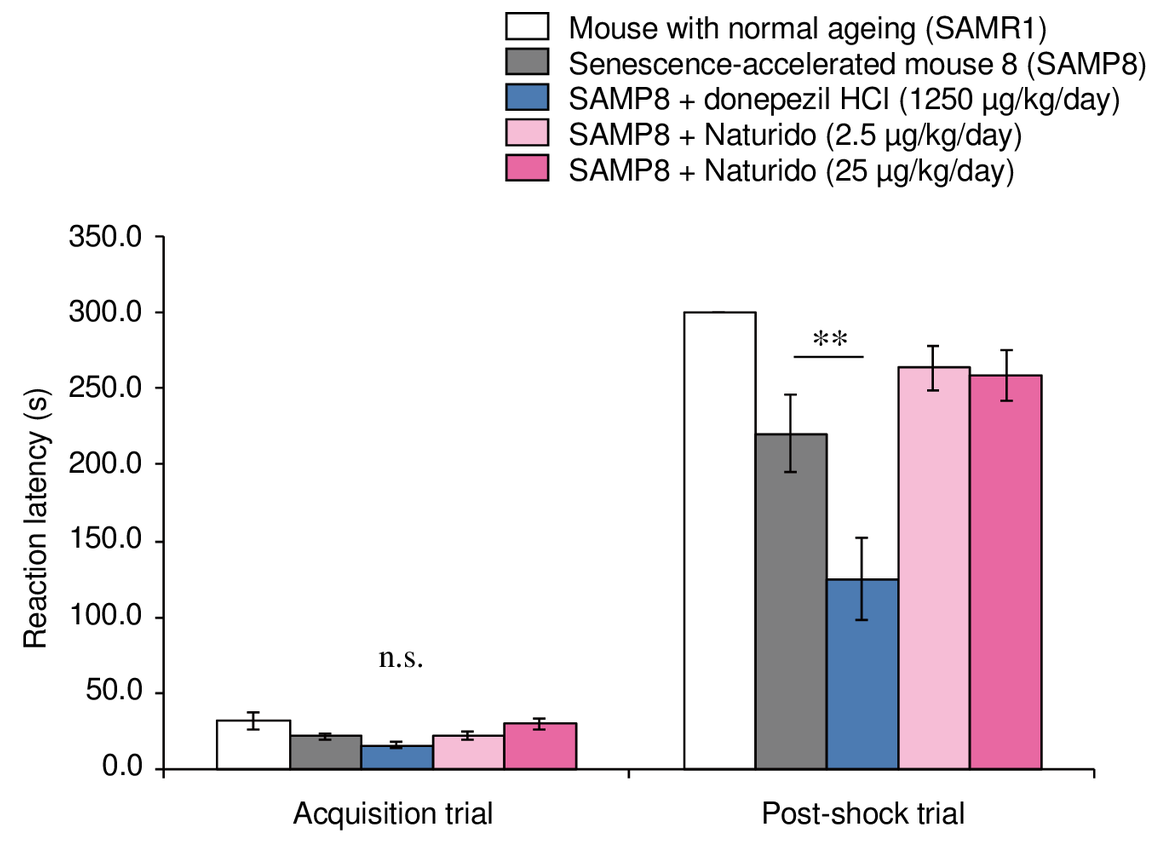

Supplement: S4 Fig — The mice were divided into five treatment groups, mice with normal ageing (SAMR1 mice) (n = 7) served as vehicle controls with oral administration of saline (0.9%) for 5 weeks, SAMP8 mice (n = 14) with oral administration of saline (0.9%) for 5 weeks, SAMP8 + donepezil HCl (1250 μg/kg/day) mice (n = 14) administered donepezil HCl (1250 μg/kg/day) orally for 5 weeks, SAMP8 + Naturido (2.5 μg/kg/day) mice (n = 16) administered Naturido orally at a dosage of 2.5 μg/kg/day for 5 weeks, SAMP8 +Naturido (25 μg/kg/day) mice (n = 15) administered Naturido orally at a dosage of 25 μg/kg/day for 5 weeks. All values are expressed as means ± SEMs. **P <0.01 vs the SAMP8 group (Dunnett test using JMP 10.0.0). (TIF) [file pone.0245235.s004.tif]

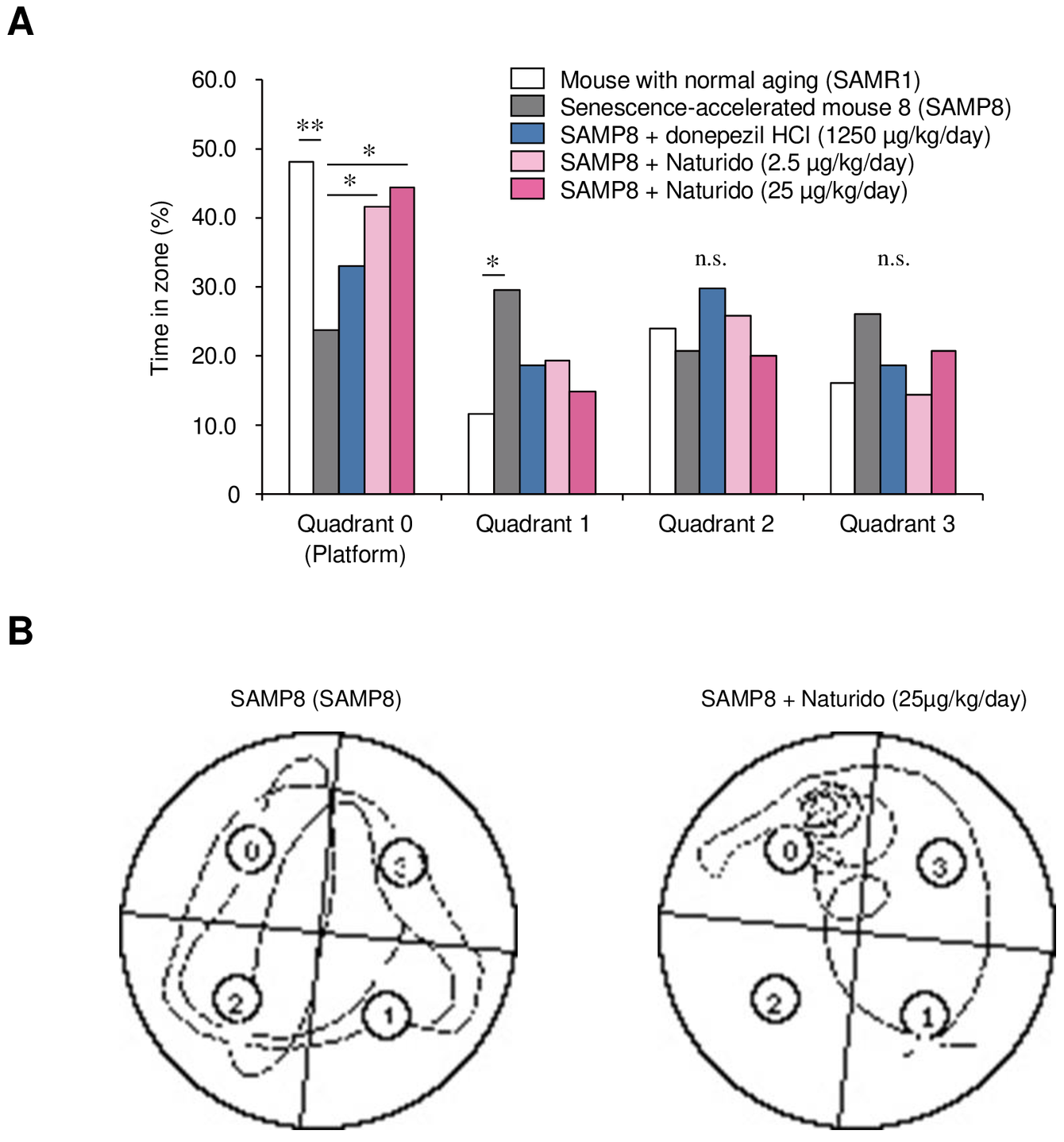

Supplement: S5 Fig — (A) The times spent in the target quadrant and in the other quadrants were compared on day 9, mice with normal ageing (SAMR1 mice) (n = 7), Senescence-accelerated mouse (SAMP8) mice (n = 12), SAMP8 + donepezil HCl (1250 μg/kg/day) mice (n = 11), SAMP8 + Naturido (2.5 μg/kg/day) mice (n = 14), SAMP8 + Naturido (25 μg/kg/day) mice (n = 7). (B) Representative images of the circular swimming path captured by video. Left, senescence-accelerated mice (SAMP8 mice); right, SAMP8 + Naturido (25 μg/kg/day) mice. All values are expressed as means ± SEMs. *P <0.05 vs the SAMP8 group (Dunnett test using JMP10.0.0). (TIF) [file pone.0245235.s005.tif]

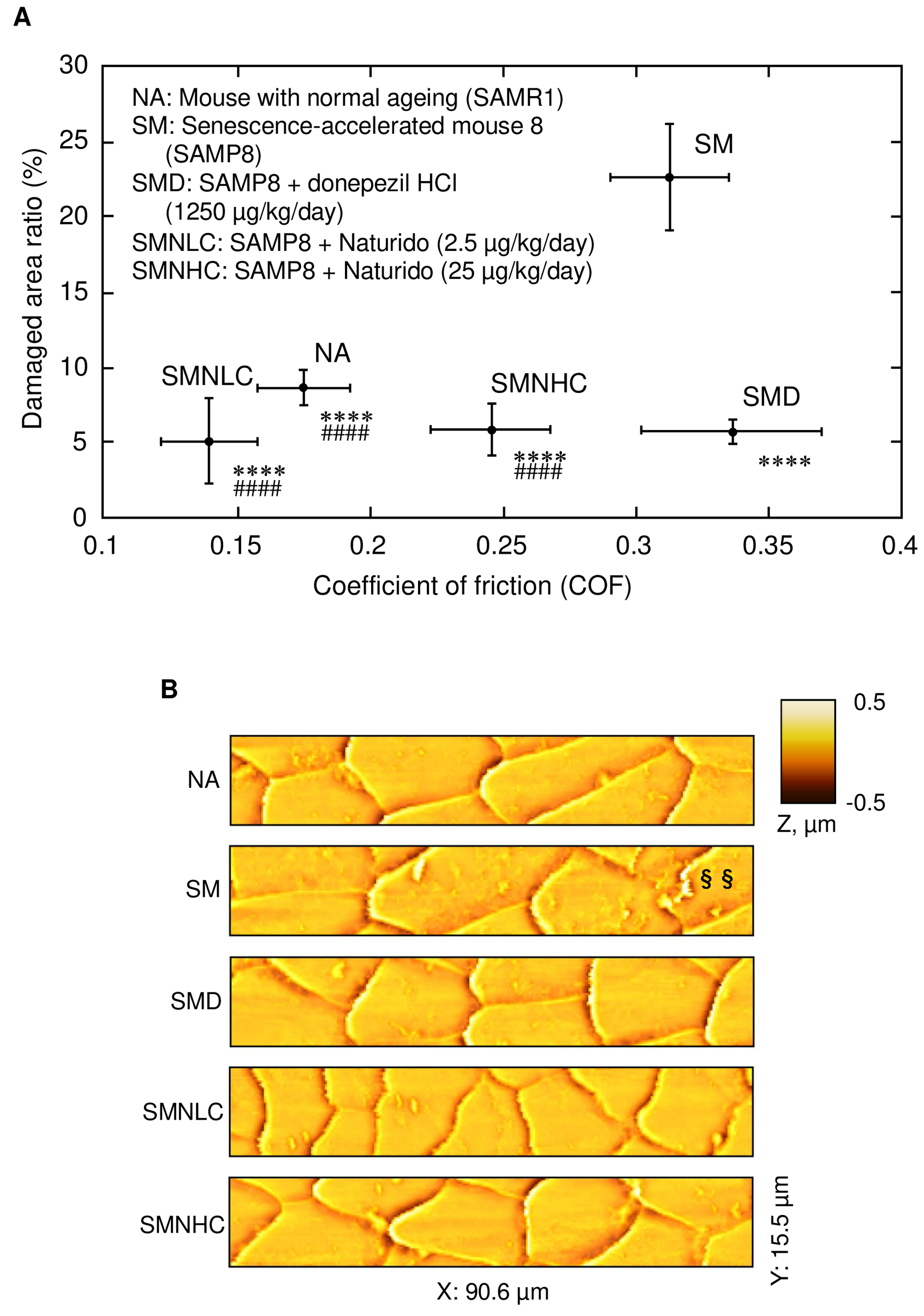

Supplement: S6 Fig — (A) Measurements (n = 5 in 5 experimental groups) were taken using a static and dynamic friction tester and a scanning probe microscope (SPM), and the COF (n = 5 in 5 experimental groups) was plotted against the damaged area divided by the total area. All values are expressed as the means ± SEMs. ****P <0.0001 and ####P <0.0001 vs the SM group (*SPM, #COF) (Dunnett test using JMP 10.0.0). (B) Representative SPM images of damaged areas in each mouse group. §A severely damaged area in an SAMP8 mouse. The upper right column shows the height of the coloured bar. (TIF) [file pone.0245235.s006.tif]

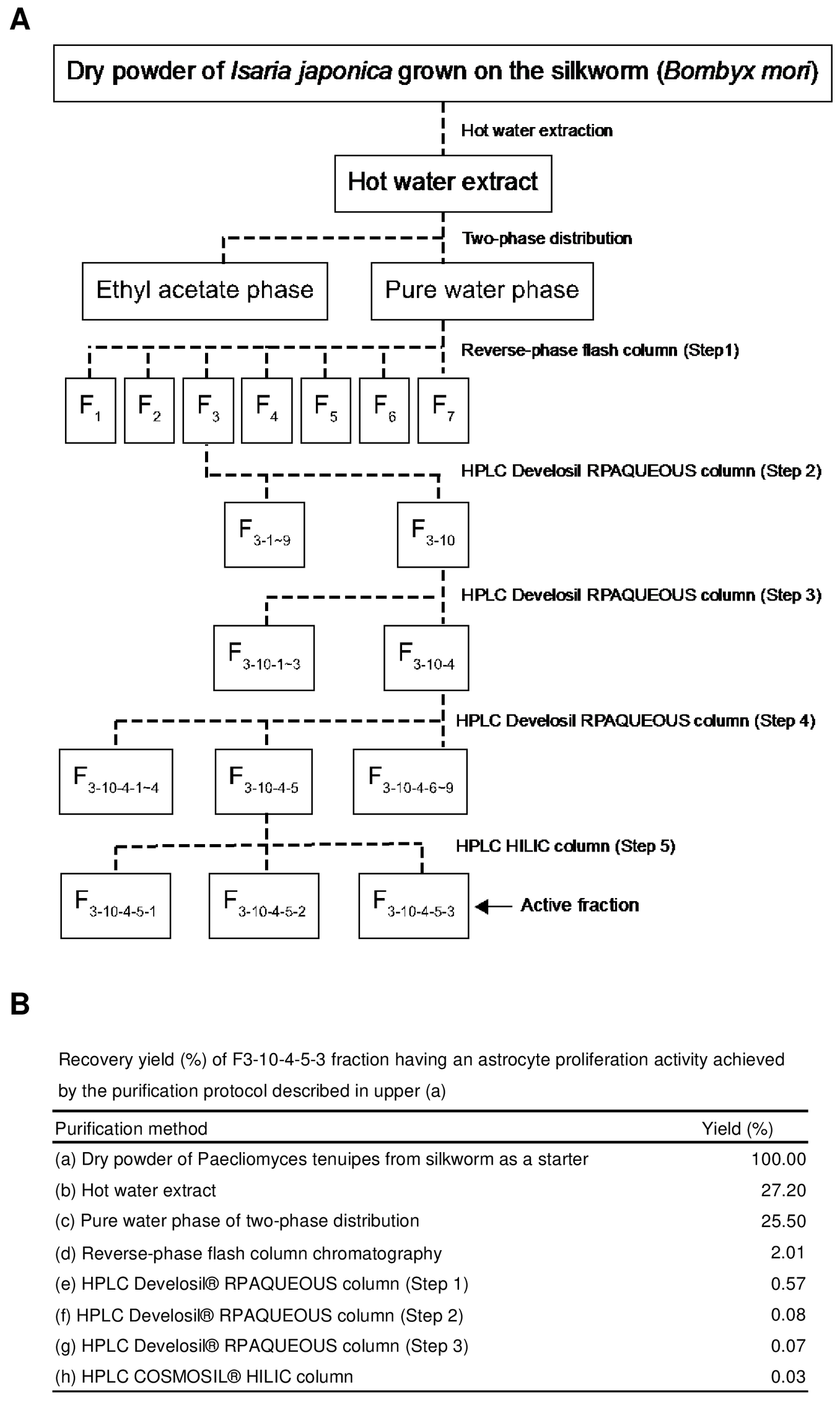

Supplement: S1 Table — (A) Beginning with 42 g of fungus powder, a purification procedure was carried out according to the flow chart, and physiological activity was tested with an astrocyte proliferation assay. F3-10-4-5-3 was confirmed as the final fraction promoting astrocyte proliferation, and the recovery of this final fraction was estimated to be 0.03% (12.6 mg) (B). Elution results from high-performance liquid chromatography (HPLC)/hydrophilic interaction chromatography (HILIC) with columns are shown in S1A Fig. (TIF) [file pone.0245235.s007.tif]

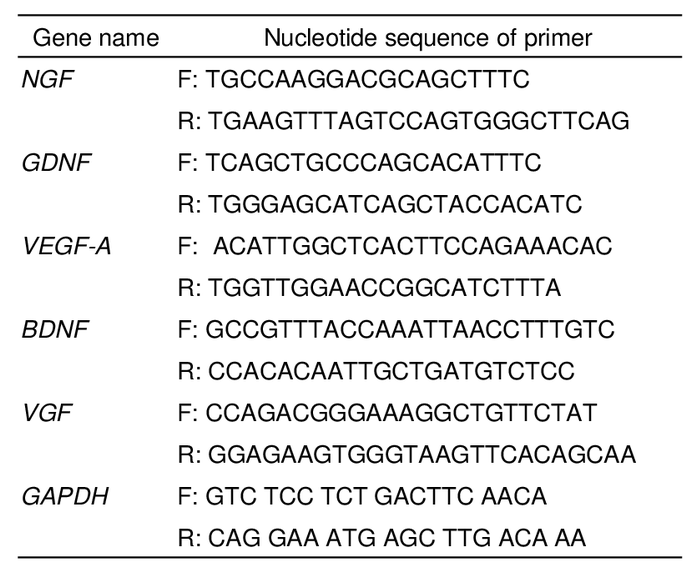

Supplement: S2 Table — Analysis of gene expression was carried out by using the synthesized cDNA as a template, commercially available primers (obtained from TaKaRa), SYBR premix Ex Taq I (TaKaRa) and a real-time PCR device, the Thermal Cycler Dice® TP800 TaKaRa). The primers comprised the following: a primer for Mus musculus Ngf transcript variant 1 mRNA (MA07578), a primer for Mus musculus Gdnf mRNA (MA102345), a primer for Mus musculus Vegfa transcript variant 1 mRNA (MA128545), a psrimer for Mus musculus Bdnf transcript variant 2 mRNA (MA138332), and a primer for Mus musculus Vgf mRNA (MA157656). The expression level of each target gene was compared after calibration against an internal standard, the housekeeping gene GAPDH. (TIF) [file pone.0245235.s008.tif]

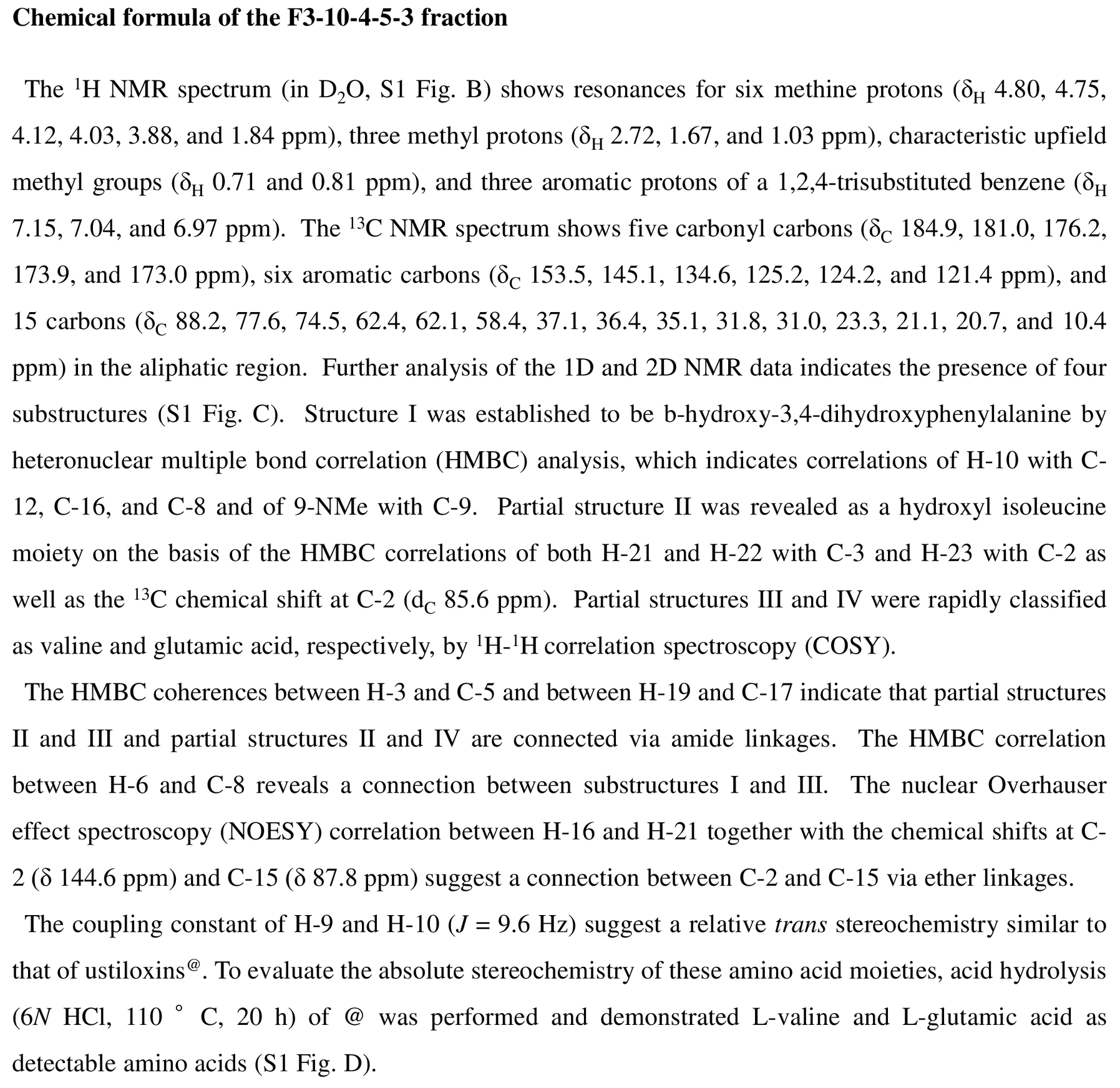

Supplement: S1 Results — (TIF) [file pone.0245235.s009.tif]
