# Supplementary material for: A novel cyclic peptide (Naturido) modulates glia–neuron interactions in vitro and reverses ageing-related deficits in senescence-accelerated mice
Source: PLoS One. 2021 Jan 27;16(1):e0245235. doi: 10.1371/journal.pone.0245235 (PMC7840003; doi:10.1371/journal.pone.0245235)
Supplement: Attachment — Submitted filename: Response to Reviewer #2.docx [file pone.0245235.s011.docx]

PONE-D-20-19724

A novel cyclic peptide (Naturido) modulates glia–neuron interactions *in vitro* and reverses ageing-related deficits in senescence accelerated mice

PLOS ONE

**Response to Reviewer #**2

We acknowledge your critical comments that encourage us to revise the manuscript.

Individual issues (in *italics*) revealed by you are treated in the revised manuscript with track changes using red colour.

1) From Line 101 (old Line 101) to Line 107: *Rephrase this sentence.*

　Your comments are very appropriate and greatly facilitate the revision of our manuscript.

--- However, the because glia–neuron interactions are extremely complex and instead of targeting individual glia or neurons as in the past, new methodologies and strategic targets should be required for the development of effective preventive and therapeutic agents against AD and other neurological disorders. In other words, innovative approaches are needed to analyse these functional complexities and to identify therapeutic neuroprotective compounds.

2) *Specify the total number of animals used and the number of animals used for each experiment.* (Please see the number of animals inserted in S1 Fig.)

　 According to your suggestions, we insert a new sentence from Line 138 (old Line 137) to Line 139 --- The applications of these mice were explained in S1 Fig. Total 75 animals were used but 15 animals that did not show any trial action were excluded in subsequent experiments.

3) *Provide No. of animals/group adopted for each technique in legends to figures.*

Your suggestions are right and important so that we have explained the number of animals/each group in the legends to S1 Fig., S4 Fig., S5 Fig., and S6 Fig.

4) *Why, is the GFAP positive cell ratio not significant in comparison to the non-treated group and control group (Figure 1C)?*

Your valuable question is very fruitful to explain not only significant function of astrocytes in this study but to highlight the role of astrocytes in diseases and thus, we exert all possible efforts to revise the sentences from Line 690 (old Line 675) to Line 707. ---

As astrocyte dysfunction is associated with dementia and brain ageing but is further important area [12], we also compared the expression of GFAP (a marker that reflects astrocyte hypertrophy) and EAAT-2 (a marker that reflects altered glia function in the studied astrocytes because double labelling shows that GFAP and EAAT-2 do not entirely overlap [31]. As a result, about 25.8 to 39.8 % of GFAP-positive cells within EAAT-2 positive cells did not change significantly upon the addition of Naturido to the astrocyte culture medium (Fig. 1C, vs before), indicating that the stability of Naturido for the GFAP-positive populations with no the loss of EAAT-2 and no the increase of GFAP [31]. In the case of another astrocyte cultures with the addition of Naturido, no fluorescence imaging using antibodies for the markers of neurons (MAP2) and microglia (CD11b /c) was observed (S3 Fig.) As GFAP is important for astrocyte–neuron interactions, and as astrocyte processes play vital roles in modulating synaptic efficacy in the CNS [32], the stable association between GFAP levels and Naturido suggests that this cyclic peptide may be useful in the analyses of astrocyte–neuron interactions. Indeed, the measurement of BrdU incorporation showed the addition of exogenous Naturido dose-dependently stimulated the proliferation of a clear proportion of astrocyte minority for GFAP and astrocyte majority for EAAT-2 (Fig. 1D), although we did not evaluate other glial populations.

5) *Why is the SEN bar absent at 0 μM (Figure 1D)?*

By your appropriate comment, we notice that Fig. 1D has two mistakes: we insert the SEM bar at 0 μM on the base of S_Source data and Statistical data and also change the vertical axis unit (200, 300, and 600 are changed to 200, 400, and 600).

6) *Do you think Naturido could be used also after pilocarpine (Vinet et al, 2018) or kainic acid (Costa eta al, 2020) induced status epilepticus, in which neurons and astrocyte are usually affected? Could Naturido be used in combination to the hydroxyprone-based inhibitor of metalloproteinase-12 to enhance its effect?*

Your excellent questions lead us to deepen a concrete consideration of the function of Naturido in practice, citing two additional references. We would like to expect your understanding for two new sentences inserted from Line 935 (old Line 912) to Line 940. ---

Recently, in the case of the pilocarpine or kainic acid-induced status epilepticus rat model, effects of metalloproteinase-12 inhibition on astrocytes, microglia and neurons in the CA3 hippocampal region are assessed and the specific metalloproteinase-12 inhibition means a potential therapeutic agent against neurological disorders [56, 57]. Naturido and the combination with Naturido and metalloproteinase inhibition also may be responsible for the treatment of status epilepsy.
